# Row crop fields provide mid‐summer forage for honey bees

**DOI:** 10.1002/ece3.8979

**Published:** 2022-06-06

**Authors:** Mary R. Silliman, Roger Schürch, Sean Malone, Sally V. Taylor, Margaret J. Couvillon

**Affiliations:** ^1^ 1757 Department of Entomology (MC0319) Virginia Tech Blacksburg Virginia USA; ^2^ 1757 Tidewater Agricultural Research and Extension Center Virginia Tech Suffolk Virginia USA

**Keywords:** *Apis mellifera*, foraging ecology, peanut foraging, row crops, waggle dance

## Abstract

Honey bees provide invaluable economic and ecological services while simultaneously facing stressors that may compromise their health. For example, agricultural landscapes, such as a row crop system, are necessary for our food production, but they may cause poor nutrition in bees from a lack of available nectar and pollen. Here, we investigated the foraging dynamics of honey bees in a row crop environment. We decoded, mapped, and analyzed 3459 waggle dances, which communicate the location of where bees collected food, for two full foraging seasons (April–October, 2018–2019). We found that bees recruited nestmates mostly locally (<2 km) throughout the season. The shortest communicated median distances (0.474 and 0.310 km), indicating abundant food availability, occurred in July in both years, which was when our row crops were in full bloom. We determined, by plotting and analyzing the communicated locations, that almost half of the mid‐summer recruitment was to row crops, with 37% (2018) and 50% (2019) of honey bee dances indicating these fields. Peanut was the most attractive in July, followed by corn and cotton but not soybean. Overall, row crop fields are indicated by a surprisingly large proportion of recruitment dances, suggesting that similar agricultural landscapes may also provide mid‐summer foraging opportunities for honey bees.

## Graphical abstract

We tracked honey bee foraging in a row crop environment using waggle dance decoding, which communicates the location of where a successful forager found food. Honey bees used these wind‐ and self‐pollinated crops for forage, especially during mid‐summer. Peanuts (yellow), corn (orange), and cotton (blue), but not soybeans (green), were attractive in July to honey bees.

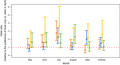

## 1 INTRODUCTION

Harvested cropland, land that includes row and sown crops, tree fruits and nuts, and vegetables, is a dominant feature in North American and European rural landscapes, covering an estimated 137 million hectares in the United States (Bigelow & Borchers, 2017). Row crop systems are largely comprised of monocultures, including corn, cotton, peanuts, and soybeans. Interestingly, although row crops are usually wind‐ or self‐pollinated, many of these crops produce pollen and nectar that attract pollinators (Gill & O’Neal, 2015; Leuck & Hammons, 1965; Martin, 1980). Previous studies have demonstrated that insect pollinators increase yield in these crops (Girardeau & Leuck, 1967; Klatt et al., 2014; Klein et al., 2007; Konzmann et al., 2019), even if they are nonessential. In soybeans, the addition of honey bee hives to the landscape increased seed number in high yield conditions (Blettler et al., 2018). Cage studies, which exclude flower‐visiting insects from blooms, showed that honey bees improve yield in some cotton cultivars, including total boll and seed mass (Rhodes, 2002). Despite these yield improvements, and perhaps because of row crop nondependency on pollinators, pollinators in row crop environments remain understudied. For example, it is unclear if these habitats can provide nutrition for pollinators: while previous studies have focused on the impact of insect pollinators to row crops, fewer studies have investigated the reciprocal relationship of the row crop habitat on the insect pollinators.

The honey bee is a valuable, managed insect pollinator, contributing c. $14–20 billion annually to the US economy (Morse & Calderone, 2000). The number of honey bee hives is decreasing in the United States, Britain, and many western European countries (Aizen & Harder, 2009a, 2009b): in the United States alone, there has been a 50% reduction in the number of hives in the last century (Aizen & Harder, 2009a, 2009b; Ellis et al., 2010; Levy, 2011; Neumann & Carreck, 2010). Honey bees use a recruitment behavior called the waggle dance to communicate the location of a good source of food, which is usually nectar or pollen (Couvillon, 2012; von Frisch, 1967). Nestmate recruits then use the waggle dance to determine the location of a profitable food source, which they can then forage on themselves. The communication therefore allows the hive to exploit the resource in an efficient and timely manner. The dance is also visible to the eye and can be observed and decoded by scientists to determine how, where, and when a forager, the dancer, is collecting food within a landscape (Beekman & Ratnieks, 2000; Carr‐Markell et al., 2020; Couvillon, Riddell Pearce, et al., 2014; Couvillon et al., 2014a, 2014b; Schürch et al., 2013; Sponsler et al., 2017; Waddington et al., 1994).

Here, we monitored honey bee foraging in southeastern Virginian agricultural land, which includes numerous corn, cotton, soybean, and peanut fields. We allowed freely flying honey bee foragers to collect nectar and pollen in a row crop environment for two complete foraging years and analyzed the honey bee waggle dances. Our objectives were: (1) to determine the communicated foraging distance, as this serves as a proxy for food availability, across the seasons within a row crop environment and (2) to calculate the percent recruitment by foragers to peanuts, soybeans, corn, and cotton. Our unique study design allowed us to observe not only where honey bees highly value forage (i.e., by decoding the waggle dance), but also to calculate how much of that recruitment occurs in fields of interest.

## 2 METHODS

### 2.1 Study organism and experimental set‐up

We studied three predominantly *Apis mellifera ligustica* honey bee colonies (labeled A–C) that included a queen and approximately 5000 workers plus brood housed in plexiglass‐walled observation hives containing three American Standard Deep frames. The observation hives were placed inside a shed (approx. 14 ft × 8 ft), and the colonies were connected to the outside via a 3 cm × 30 cm plastic tube, which afforded the foragers unimpeded access to the landscape. We wired the shed for electricity to provide diffuse lighting and, when needed, air conditioning or heating. Because observation hives are small and the bees cannot maintain large honey stores (i.e., sufficient for a week of bad weather), we provided when needed a sugar solution for supplemental feeding using a canning jar with small holes in the lid inverted over a mesh opening in the observation hive.

### 2.2 Study location, crops, and bloom times

We located the colonies at the Virginia Tech Tidewater Agricultural Research and Extension Center (TAREC) (36°41′06.4″N 76°46′01.6″W) in Suffolk, Virginia. The 465‐acre facility researches southeastern Virginia row crops (e.g., corn, cotton, peanuts, soybeans, and small grains) and swine. The TAREC comprised the immediate area around the honey bee hives, but within a wider foraging range (approx. 8.3 km radius) there was a mixed‐use landscape of forests (33.5%), cropland (30.4%), wetlands (18.75%), developed land (4.9%), grassland/pasture (3.55%), and other land types (8.9%) (USDA‐NASS 2018, 2018; USDA‐NASS 2019, 2019). Because there were unrealistic fluctuations from year to year in the CropScape land type characterizations, and because some of the land types in our crops of interest were demonstrably false (i.e., we had first‐hand knowledge that a particular field in TAREC was, for example, one crop whereas it was mislabeled as another in CropScape), we manually mapped and digitized the crops of interest within the region where most of the foraging occurred. Our crops of interest were peanuts, soybeans, corn, and cotton, which accounted for 34–36% of the landscape around the hives within a 2 km radius in 2018 and 2019 (Table 1; Figure 6).

**TABLE 1:** Percent of crops of interest within an area that represents the majority of dances (i.e., within a 2.0 km radius form the hives). Land‐type percentages are from the (*manually corrected*) 2018 and 2019 National Cropland Data layers and therefore are subject to slight yearly variations

	2018	2019
Corn	8.358	11.2
Cotton	6.841	10.57
Peanut	5.528	6.078
Soybean	15.16	5.862
other	64.11	66.28

**FIGURE 1:**
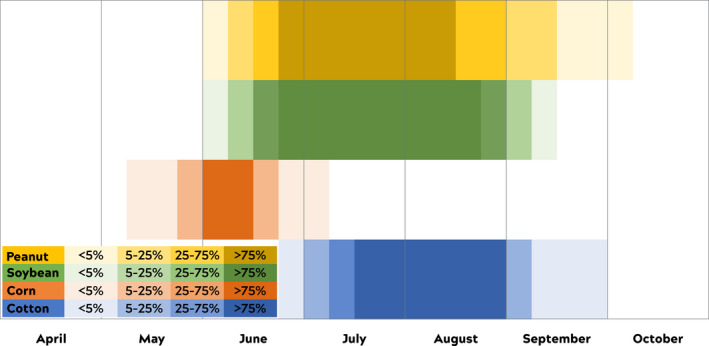
Approximate bloom times of row crop cultivars in Suffolk, Virginia. Bloom intervals are divided based on the percentage of bloom (i.e., >75% bloom refers to greater than 75% of fields of interest in bloom). We investigated percent honey bee foraging in fields of interest when >75% of the crop of a field was actively flowering

Row crops vary in their bloom times (Figure 1). Small grains and cover crops (i.e., crops planted for a variety of beneficial effects that are not harvested and sold as the main cash crop in an operation) bloom from early April to mid‐May. Major row crops begin to bloom around mid‐June to early July. For this study, we were interested in examining honey bee foraging on peanuts, soybeans, corn, and cotton because they all bloom around the same time, beginning between mid‐June to early July with the bloom season lasting until mid‐ to late‐August. Additionally, it is often anecdotally assumed that bees prefer soybeans to cotton, but, to our knowledge, it has never formally been investigated. In our calculations of percent foraging of specific fields of interest (see below), we restricted the date ranges to the time intervals when c. 75% of the crop is in bloom, which we consider full bloom (Figure 1).

### 2.3 Data collection—waggle dance filming

Foragers may perform waggle dances to recruit nestmates to high quality resources, generally 100 m to many kilometers from the hive (von Frisch, 1967). Importantly, foragers only dance for the best resources at any given time (Couvillon, 2012; Couvillon & Ratnieks, 2015; Seeley, 1994), and this allows us to determine the location of where the bees are most preferring to forage, not the location of all the available forage including low quality forage. When we decode waggle dances, we do so because we are interested in where the dancer herself has foraged: by definition, every dancer is (1) a recently returned forager who (2) has found a highly profitable resource, usually nectar or pollen. We, the eavesdropping scientists, capitalize upon the dance communication that she is doing to know where she has foraged.

We filmed each colony for about an hour per day (~9:30 am–12:30 pm, but usually 10–11 am), 4–5 days per week using Canon Vixia HF R82 cameras. Videos were recorded to SanDisk Extreme SD cards and uploaded to a Google Team Drive (GTD) for dance decoding (see below). Filming was conducted on days with a minimum temperature of 14°C and no rain or adverse weather. Plumb lines (fishing wire suspended from the top of the observation hive with a push pin with a washer or bolt at the bottom) spaced 5 cm apart were used as guides to adjust cameras so that approximately 20–25 cm of the dance floor was visible. Prior to filming, we recorded the date, time, hive identifier, blooming crops and weeds, and outside temperature. We filmed during the entire foraging season for two years (23 April–31 October, 2018 and 10 April–18 October, 2019). We generated 1‐h long video per day from each of the three observation hives, usually 4 days per week and 4 weeks per month, for six and a half months per year and for 2 years to make approximately 624 h of video.

### 2.4 Data collection—waggle dance decoding

Dance decoding was based on an updated version of Couvillon et al. (2012). Briefly, we converted video files filmed at 30 frames per second to AVI using Ubuntu (v. 2004.2021.222.0) and imported them into ImageJ (version 1.52i). At the start of each video, we measured our vertical reference plumb line to determine the angle offset (i.e., if the cameras and/or observation hive are not completely vertical, then measuring the angle offset corrects for that, usually only by a few degrees or fractions of a degree). Then we played the video from the beginning until we found the first dancer, which usually occurred within the first few seconds of the video. A returning forager performing a waggle dance will repetitively oscillate her abdomen while moving linearly (waggle run phase) across the comb, will stop, turn either to the left or right, and then walk back (return phase) to the starting position. She may then do another waggle run, repeating this pattern of waggle run and return phase 1–100+ times (Seeley et al., 2000; von Frisch, 1967).

Dance decoding involves extracting two pieces of information per waggle run to obtain a vector: the duration of the run and the direction of the run relative to vertical (Couvillon, Riddell Pearce, et al., 2012; von Frisch, 1967). We assessed the duration by noting the start and end frame of each waggle run and calculating the difference, which we then convert from number of frames to seconds of duration by dividing by the 30 fps. We assessed the direction of each waggle run by using the straight‐line tool in ImageJ to measure the angle from vertical that the dancer makes with her body as she performs a waggle run (Couvillon, Riddell Pearce, et al., 2012; von Frisch, 1967). This measurement is automatically corrected for the angle offset (see above) in Excel. Lastly, since honey bees orient relative to the sun but dance relative to vertical, we noted the time of day and date during filming and corrected later for the solar azimuth to obtain the bearing.

For each dance, we decoded four mid‐dance, consecutive waggle runs per dancer, with an equal number of left and right‐handed turns (Seeley et al., 2000; von Frisch, 1967). We averaged the four waggle run durations and azimuth‐corrected angles to obtain a single duration and direction for each dance. This number and selection of waggle runs was chosen because the calculated mean direction and mean duration was not significantly different from the means obtained if all the waggle runs per dance are decoded (Couvillon, Riddell Pearce, et al., 2012). We converted duration to distance using the universal calibration reported by Schürch et al. (2019). Taken together, these averaged components (direction +distance) give the approximate location of the advertised resource.

Once the first dance within that video frame had been decoded, we then decoded the other dances that were occurring simultaneously on the dance floor. In videos with few dancers present, we watched the entire video and decoded every dance. However, in videos with higher activity (20+ per hour), we skipped ahead 6 min (or 10,789 frames) after decoding all the simultaneous dances that occurred at the start of the video. By skipping ahead, we avoided potential pseudo‐replication, where we sample from the same dancer twice [i.e., a single dancer can dance 1–100 times (Seeley et al., 2000), depending on the resource quality, which can last many minutes and can involve the dancer moving in and out of the screen]. Our objective was to achieve at least 20 dances per hour of video. These dances should provide a good representation of colony foraging during the respective hour of filming (Couvillon, Riddell Pearce, et al., 2012; Sponsler et al., 2017). In all, we decoded 3459 dances, 2066 from 2018 and 1393 from 2019. We used R (R Core Team, 2020) to perform all the analyses.

### 2.5 Data management and validation

For waggle dance decoding, we recorded all raw video data on SD cards, which were subsequently uploaded to a GTD as a permanent, online, cloud‐based repository. We immediately entered decoded dance data into Excel, and each decoder would upload his or her Excel file daily to the GTD. To avoid version confusion, the latest raw data as entered the Excel files were obtained from the GTD through R code using the googledrive package. Lastly, we created a GitHub repository to manage R files.

We validated dance data before analysis. Data were scanned and rows with missing values, due to human error, were removed. Next, we examined our data for erroneous values by calculating, for each dance, the standard deviation (SD) per dance for each dance component (i.e., the SD between the four runs for waggle run duration and for waggle run angle). We then identified the highest ten values (i.e., the dances with the most intra‐dance variation) within the data set for each respective dance component (i.e., duration, angle). We located these 20 dances and examined them on the data entry sheet to determine if the SD was due to an obvious entry error. If the error source was unclear, we located the dances on the original videos to verify whether high SD was based on error inherent in the dance (i.e., a “noisy” dancer with high intra‐dance variation), which is normal (Couvillon, Phillipps, et al., 2012; Couvillon, Riddell Pearce, et al., 2012; Schürch & Couvillon, 2013; Schürch et al., 2019) and acceptable, or due to human error (i.e., dance decoding error), in which case we re‐decoded the dance. We noted any changes to initial values in our dataset. The process of examining the 10 dances with the highest SD values in either duration or angle was repeated until all 10 dances’ SDs were solely from error inherent in the dance (i.e., were just noisy dances). Overall, this required us to validate six rounds of highest SD value dances for both duration and angle and involved our correcting (re‐decoding) 30 dances out of a dataset of 3459 dances.

### 2.6 Data analysis—distance

Honey bees have evolved to be economic in their foraging decisions and, considering the energetic impact of a flight, a bee will only fly and then recruit her nestmates to a resource as necessary, especially given the distance from the hive (Couvillon et al., 2014b; Schmid‐Hempel et al., 1985; Seeley, 1986). Therefore, communicated foraging distance can serve as a proxy for the availability of nectar and pollen (i.e., when bees recruit for a resource relatively further away, it is likely because food cannot be found closer to the hive).

We interpreted the communicated foraging distance as a function of time to determine seasonal fluctuations in forage availability. We began by plotting the raw data as distance across time, which included a LOESS regression. We then aggregated time by month, which allowed us to compare median foraging distance per month to previous studies (Couvillon et al., 2014b). Lastly, because our study was in an environment with semi‐predictable bloom times highly relevant to our study, we aggregated median foraging distance across the growing season (e.g., pre, during, and post bloom) by crop. We expected to see communicated foraging distance decrease as we go from pre‐ to during bloom and increase as we go from during bloom to postbloom if the crop is impacting food availability.

### 2.7 Data analysis—percent recruitment by foragers and mapping in fields of interest

We were primarily interested in honey bees’ use of soybean, cotton, corn, and peanut fields (Figure 1, Table 1) because of our regional land cover, the timing of the crops, and the gaps in the previous research. Honey bees are imprecise and inaccurate in their dance communications and there is an inherent error in both components of the dance (Okada et al., 2014; Schürch & Couvillon, 2013; von Frisch, 1967). Therefore, predicting and mapping waggle dances as exact foraging locations, as was done in the past (Beekman & Ratnieks, 2000; Steffan‐Dewenter & Kuhn, 2003), over‐represents our certainty. Instead, our methodology where individual dances are decoded, simulated, and then mapped as a probability cloud, considers the error in the dance.

We used the methodology described in Schürch et al. (2013, 2019) to obtain a probabilistic prediction of foraging location. In both years, 95% of foraging was just within 2 km (1.921 km and 1.895 km for 2018 and 2019 respectively), and we therefore constrained our region of interest for visitation to crop of interest to 2 km. Within the 2 km, we traced manually the fields surrounding TAREC in Google Earth, and we then assigned each the proper crop for the year. The resulting KML file were imported into R (R Core Team, 2020), where we then simulated each observed dance's advertised location 1000 times. For each dance, we then picked one prediction and calculated the percentage of dances in fields of interest. This procedure was repeated 10,000 times, each time with different combinations of predictions from each dance and resulting in a distribution of 10,000 percentages. From the distribution of percentages, we then took the median percentage as our point estimate and the 2.5th and 97.5th percentile for our confidence interval.

Using this procedure, we calculated percent recruitment by the foragers for pre‐, during, and postbloom per crop to understand how recruitment (high foraging interest) in our fields changes throughout the season and with crop bloom. We binned the landscape in a 25 × 25 m grid and calculated the percentages of recruitment dances pointing to each grid square during the designated time periods (prebloom, bloom, postbloom) and overlaid the dances on the land‐use map. Transparency denotes high interest, where the greater the visibility of the colors (black as nonrow crop fields, colors as row crop fields), the more that area was indicated by a waggle dance.

### 2.8 Data analysis—determining relative attractiveness of row crops

Lastly, we wished to compare the relative attractiveness of the row crops with each other, a calculation that requires we correct for distance (Couvillon et al., 2014a). To do this, we performed a multinomial logistic regression, where the response variable was whether a dance indicates a particular row crop versus other (reference category) field, which we determine against the predictor variables of month (reference category: April) and distance. We included year as a random factor. To deal with the uncertainty in our observations, we, similar to our calculations of percent recruitment by foragers to fields of interest, repeated a thousand models, each time taking only one simulated dance per observed dance. From these 1000 model runs, we calculated the point estimate (the odds ratios) again as the median of the estimated coefficients, and the 2.5 and 97.5 percentile for lower and upper confidence bounds.

All data and code are available to the scientific community in a permanent data repository at Virginia Tech (https://doi.org/10.7294/19755016).

## 3 RESULTS

### 3.1 Honey bees in row crops recruit mostly locally with some long‐range events

**FIGURE 2:**
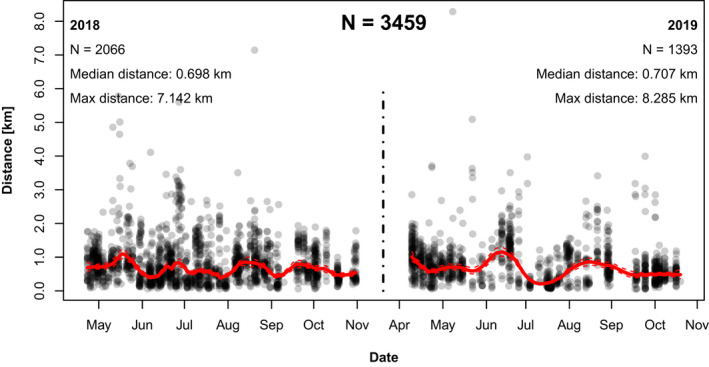
Honey bees in row crops foraged mostly locally with some long‐range events. Communicated distance travelled by foragers (km) throughout the foraging season in 2018 and 2019, Suffolk, Virginia. Raw data is fitted using LOESS regressions (span = 0.1). Each point is a single decoded dance

The median distance foragers recruited to in the landscape during the study (April–October, 2018 and 2019) was 0.702 km (*n* = 3459) with a range from 0.050 to 8.285 km (Figure 2). The median distance was 0.698 km (range 0.058–7.142 km) (*n* = 2066) and 0.707 km (range 0.051 to 8.285 km) (*n* = 1393) in 2018 and 2019, respectively. The maximum distance in 2019 (8.285 km) was larger than in 2018 by over 1 km.

### 3.2 Foraging distance varies by month and year

**FIGURE 3:**
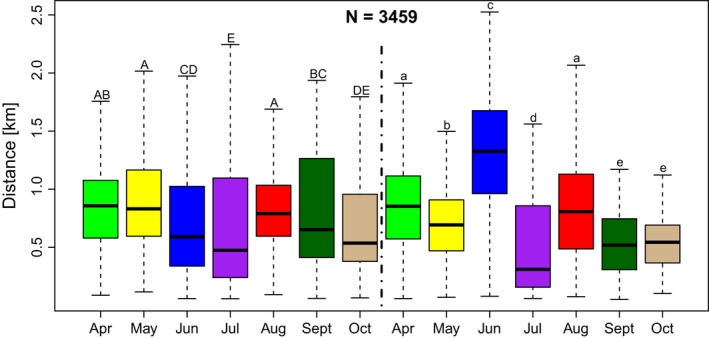
Communicated foraging distance, which indicates food availability, varies by month and year. Median foraging distance travelled (km) by month (*n* = 3460) in 2018 and 2019, Suffolk, Virginia. Months with different letters are different (*p* < .05) in post hoc comparison. Post hoc results are capitalized for year one and lowercase for year two and results show comparisons between the months in each respective year

Foraging distance, as communicated by waggle dancers, varied by month in both years (2018: χ^2^ = 104.65, df = 6, *p* < .0001, *n* = 2066; 2019: χ^2^ = 275.71, df = 6, *p* < .0001, *n* = 1393; Figure 3). In 2018, the highest median communicated foraging distances were in April (0.857 km), May (0.831 km), and August (0.790 km), suggesting these are months when forage is less available. In 2019, the highest median communicated foraging distances were in April (0.853 km), June (1.326 km), and August (0.807 km). June 2019 was the highest median communicated foraging distance for both years, which we suspect was driven by drought (see Discussion). The lowest communicated median foraging distances were observed in July in both years, which suggests that forage is most available at this time. July is the month when all target row crops are blooming (Figure 1).

### 3.3 Communicated median foraging distance varies during crops’ full bloom

**FIGURE 4:**
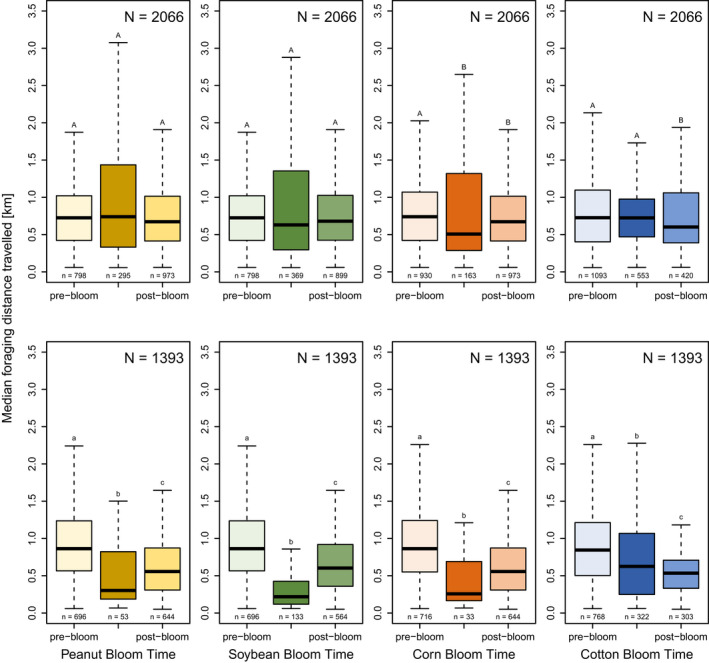
The median foraging distance, which is a proxy for available forage, is variably driven by bloom. Median foraging distance travelled (km) by bloom time per crop in 2018 (*n* = 2067) and 2019 (*n* = 1393). Distance travelled during pre‐bloom, bloom, and post‐bloom are shown based on bloom intervals for each crop (e.g., peanut, soybean, corn, cotton). Bloom signifies full bloom, when >75% of fields are in bloom. Post hoc results are between the bloom intervals for each crop by year. 2018 post hoc results are capitalized and 2019 results are lowercase. Colors correspond to the crop bloom times from Figure 1

The median foraging distance travelled during pre, during, and post bloom followed our prediction for corn (2018, 2019), peanut (2019), and soybeans (2019) (Figure 4). Cotton (2019) did demonstrate a decrease in distance during bloom, but postbloom decreased even further, presumably because additional forage became available closer to the hive (see Discussion).

### 3.4 Honey bees recruit to row crop fields during the mid‐summer

**FIGURE 5:**
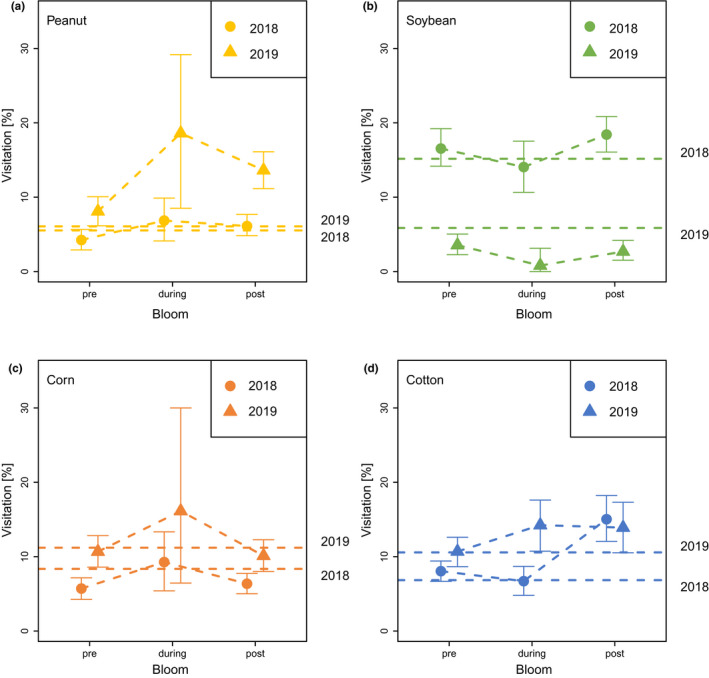
Honey bees increase recruitment to peanut and also corn and, to a smaller degree, cotton during bloom but not soybean. Mean percent (95% CI) foraging in peanut (yellow), soybean (green), corn (orange), and cotton (blue) fields during bloom intervals (pre‐bloom, during bloom, post‐bloom) in 2018 (circle) and 2019 (triangle) when >75% of the fields of interest are in bloom. Dashed lines represent the percent land cover (95% foraging range) in 2018 and 2019 of that row crop. Visitation [%] refers to the dancer, not the recruits

**FIGURE 6:**
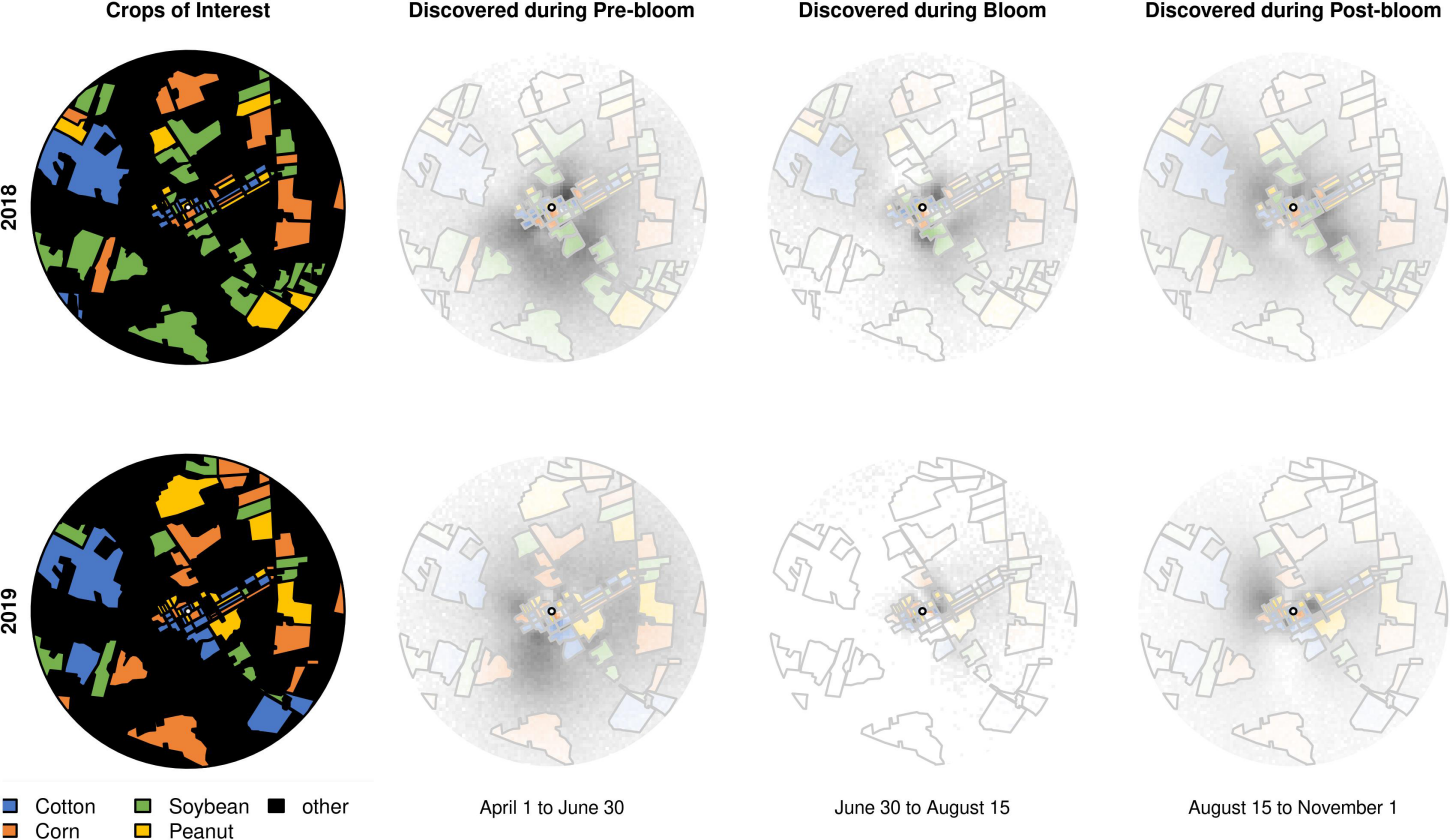
Honey bee dancers indicate the row crops during bloom compared to pre‐ and post‐bloom. Transparency denotes level of recruitment, where increased color indicates higher recruitment from foragers. In particular, the large black patches that are not row crops are visible in pre‐ and post‐bloom. During bloom, the transparency moves inwards to focus on the colorful row crop fields

Percent recruitment by foragers to fields of interest [per crop] increased from pre‐bloom to bloom, as we would expect if bees were foraging in the crops, in peanuts (2018 and 2019) and corn (2018 and 2019) and in cotton (2019), especially if one considers the representation of these crops (%) in the landscape (Table 2, Figure 5). This increase was most evident in peanut and in corn in 2019. For example, peanuts accounted for approx. 6% of the landscape within their immediate foraging range for both years, and yet the bees foraged upon and recruited to peanuts 7% and 19% in 2018 and 2019, respectively, during bloom. This means that bees used peanuts during bloom time 3–5× more than one would expect given its representation in the landscape in 2019. The bees’ indication through the waggle dance of the peanut fields also followed the predicted down‐up‐down pattern that one would expect if the foraging were driven by that crop's bloom (Figures 5 and 6).

**TABLE 2:** Mean percent foraging (95% CI) in fields of interest by bloom in 2018 and 2019, as determined by waggle dance decoding, mapping, and analysis. During bloom is the period when >75% of fields are in bloom. Pre‐ and post‐bloom periods comprise percent foraging in fields of interest before and after the period of full bloom. Percent land cover displays the proportional representation of that land type within the landscape

	Year	% Landcover	Pre‐bloom	During	Post‐bloom
Peanuts	2018	5.5	4.2 (2.9–5.7)	6.9 (4.1–9.9)	6.1 (4.8–7.7)
Peanuts	2019	6.1	8.1 (6.2–10.1)	18.6 (8.5–29.2)	13.6 (11.1–16.1)
Soybeans	2018	15.2	16.5 (14.2–19.2)	14.1 (10.6–17.5)	18.4 (16.1–20.8)
Soybeans	2019	5.9	3.5 (2.3–5.0)	0.8 (0.0–3.1)	2.7 (1.5–4.2)
Corn	2018	8.4	5.7 (4.3–7.2)	9.3 (5.4–13.3)	6.4 (5.0–7.8)
Corn	2019	11.2	10.7 (8.6–12.8)	16.1 (6.5–30.0)	10.1 (8.0–12.3)
Cotton	2018	6.8	8.0 (6.7–9.4)	6.7 (4.8–8.7)	15.0 (12.1–18.2)
Cotton	2019	10.6	10.7 (8.7–12.6)	14.2 (10.7–17.6)	13.9 (10.5–17.3)

In contrast, bees decreased their recruitment to soybeans from pre‐bloom to bloom in both years and to cotton in 2018 (Table 2, Figures 5 and 6). Soybeans accounted for 15% and 6% of the landscape in 2018 and 2019, and the bees foraged upon and recruited to the soybean fields during bloom 14% and then 1% respectively, with both percent recruitment decreasing pre‐bloom to bloom. In other words, not only was soybean not driving foraging, but the bees decreased their recruitment to the soybean fields during bloom, where the mean % recruitment during bloom for both years was lower than the lower CI value in pre‐bloom (Table 2, Figure 5 and 6). The bees’ indication through the waggle dance of the soybean fields was opposite of peanut and instead followed an up‐down‐up pattern for both years.

Corn also was an attractive foraging option for honey bees during the mid‐summer. Corn accounted for 8% and 11% of the landscape, and approximately 9% and 16% of recruitment indicated the fields during bloom (Table 2, Figures 5 and 6). For 2018 and especially for 2019, the dancing for corn increased preboom to bloom and decreased again in postbloom (down‐up‐down).

Lastly, cotton was variably attractive to foraging bees: in 2018, while the fields accounted for 7% of the landscape within the foraging range, the bees indicated the fields the most in the post bloom period (15%), whereas their recruitment to those fields in the pre bloom period (8%) and bloom period (7%) did not increase (Table 2, Figures 5 and 6). In 2019, while the fields accounted for 11% of the landscape, the bees indicated the fields the most during the bloom (14%), which was an increase from pre bloom (11%).

As these row crops bloom over roughly the same period, taken together, the row crops account for a large proportion of foraging during full bloom (c. 37–50% in total, Table 2, % foraging during bloom), representing a large effort by honey bee hives in exploiting row crops for forage.

### 3.5 Honey bees recruit in July especially to peanuts and also to corn and cotton across entire study

**FIGURE 7:**
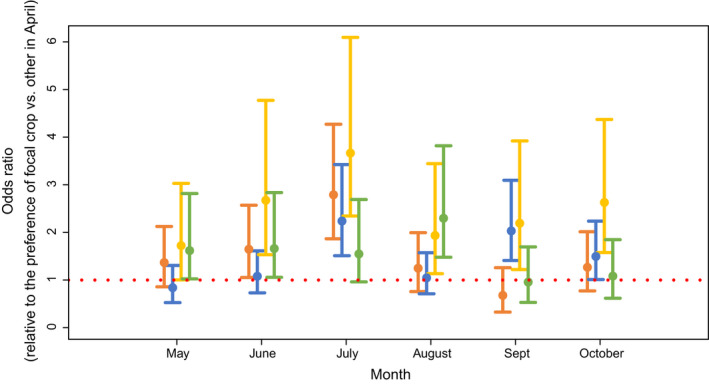
Honey bees indicate peanut (yellow) the most in July compared to other fields and compared to April. Corn (orange) and cotton (blue), but not soybean (green), are also highly attractive. Odds ratio of crops displaying relative attractiveness by month with distance correction. Soybean did demonstrate peak recruitment in August, and cotton showed a second peak in attractiveness in September. Year included as a random factor

After we correct for distance, honey bee dances indicated the highly attractive peanut fields the most in the month of July compared to other fields and compared to April [Multinomial logistic regression, Odds ratio (OR, 95% CI): 3.7 (2.3–6.1), 2018 and 2019]. Corn [OR 2.8 (1.9–4.3)] and cotton [OR 2.2 (1.5–3.4), Figure 7] were also highly attractive. In contrast, soybean fields were not indicated by waggle dances more compared to other fields [1.5 (1.0–2.7), Figure 7]. Recruitment to soybean fields does increase in August [OR 2.3 (1.5–3.8), Figure 6] relative to the other crops. In August, soybeans are still blooming, but corn is largely finished (Figure 1). Similarly, cotton also displayed a second increase in attractiveness in September [OR 2.0 (1.4–3.1), Figure 7], which is well past when the other row crops are blooming.

## 4 DISCUSSION

Here, we have shown that honey bees in an agricultural landscape forage extensively in nearby row crops, especially during the mid‐summer full bloom period. Recruitment was generally local, <2 km away from the hive throughout the foraging year (April–October) (Figure 2). We found some variation per month, with communicated foraging distance increasing (i.e., forage availability decreasing) in April, May, June, and August (Figure 3). The month where distance was shortest, indicating that food was most abundant, was observed for both years in July, the only month when all target crops (peanuts, soybeans, corn, cotton) were in full bloom (Figure 1). Likewise, our calculations of the % recruitment to fields of interest demonstrate that corn, cotton, and especially peanut are playing a significant role in honey bee foraging during mid‐summer, with dancing honey bees indicating row crop fields for nearly half of their dances (Table 2, Figures 5 and 6). Only soybean bloom did not cause an increase in recruitment (foraging) to those fields. Lastly, by correcting for distance and analyzing dance preference stratified by crop and month, we demonstrated that peanuts, followed by corn and cotton (but not soybean) is most indicated in July compared to other fields (Figure 7). Taken together, all these data suggest that row crops provide abundant forage for honey bees during the mid‐summer, a period that has previously presented as a time of forage dearth in other landscapes (Couvillon et al., 2014b; Couvillon, Fensome, et al., 2014).

Other studies have investigated honey bee foraging dynamics in row crops, with the majority focusing on the pollination services bees provide to crops (Blettler et al., 2018; Erickson, 1975; Girardeau & Leuck, 1967; McGregor et al., 1955; Pires et al., 2014). Row crops are wind or self‐pollinated and do not depend upon insects for successful pollination; however, they have flowers that produce nectar and pollen and, in the case of cotton, extrafloral nectaries that attract pollinators and provide forage (Martin, 1980; Röse et al., 2006; Willmer, 2011). Our study demonstrates that, when placed in a row crop environment, honey bees will forage and recruit to the row crops, primarily during the periods of full and, sometimes, postbloom (Figure 6, Table 2). Percent recruitment during full bloom was especially high in 2019 to peanut (19%), corn (16%), and cotton (14%). Because we possessed first‐hand knowledge of field locations, we also anecdotally could see how crop rotation might affect bee recruitment choices: in 2019, some peanut fields were located closer to the hive, which could explain the large increase in the use of those fields. Of course, though, our distance correction still demonstrated peanuts as the most preferred crop (Figure 7).

During bloom in July, while peanut, corn, and cotton accounted for c. 28% of the landscape within the foraging range, the honey bees indicated these fields c. 49%. Therefore, dancing bees are recruiting to the row crops nearly twice as much as one would predict based on how prevalent the fields are in the landscape and their distance from the hive. Additionally, communicated foraging distance serves as a proxy for forage availability, where high availability of forage results in a lower communicated foraging distance and lower availability of forage results in higher communicated foraging distance (Couvillon et al., 2014b). The communicated foraging distance for peanut and corn in 2019 is low (Figure 4), supporting that the foraging bees are finding abundant resources close to the hive. In contrast, soybean was not appreciably indicated by our recruiting bees, with the bees use of these fields decreasing in the bloom compared to the pre‐bloom, which is also reflected in the noneffect of bloom on the foraging distance (heavily overlapping CIs, Figure 4).

Interestingly, honey bee recruitment increased to cotton in 2018 and to soybean in 2018 and 2019 from bloom to postbloom, which might at first seem counterintuitive. There are two potential and nonmutually exclusive explanations for these data. Bees will increase the use of a field from bloom to post bloom if something attractive starts to flower within the field in the post bloom period, such as the emergence of blooming weeds following final herbicide applications. Additionally/alternatively, cotton and peanut possess indeterminate flowering habits, where blooms are often available in the post bloom period if the climate is favorable and plants have not reached physiological maturity, although the percentage of blooming plants is usually <75% (Quisenberry & Roark, 1976; Ritchie et al., 2007). Cotton was relatively more attractive again in September (Figure 7), which could suggest the bees return to the remaining cotton blooms when the other crops have ceased blooming but before autumn forage, like goldenrod, becomes available (Richardson et al., 2015). One limitation of using the dance language alone is that we cannot immediately test between these alternative explanations.

Our multinomial logistic regression analysis, which corrects for distance, demonstrates that honey bees possess a strong preference for peanut, then corn and cotton, fields. In other words, dancing honey bees are 2–3 times more likely to recruit to peanut, corn, or cotton fields in July compared to other fields and compared to that preference in April (Figure 7). In contrast, peak soybean recruitment happened in August, which does still fall within their bloom time (Figure 1). Soybeans do continue to bloom into mid‐August (Figure 1). Therefore, like the September cotton peak, this small increase might also represent the bees demonstrating a renewed interest in soybean once the more attractive peanut and corn are past their peak blooms.

Previous investigations have used our waggle dance decoding and mapping methods (Balfour & Ratnieks, 2017; Bänsch et al., 2020; Carr‐Markell et al., 2020; Garbuzov et al., 2015; Sponsler et al., 2017), demonstrating its wide suitability for determining how honey bees use particular landscapes, fields, or flora for food. Overall, how honey bees use a landscape is a complex interaction of colony requirements, season, distance to field/crop of interest, and availability of alternative forage. In the springtime, when abundant alternative forage will be present, honey bees visit oilseed rape fields 0–26%, depending on whether the hives were in an urban or rural location respectively (Garbuzov et al., 2015). In our study location, at nearly 50% of all foraging in mid‐summer was in row crops, especially peanut, corn, and cotton. Cotton, soybean, and peanut produce both nectar and pollen, and cotton secretes nectar through extrafloral nectaries; however, these are not usually viewed as high quality food sources (Martin, 1980). Indeed, our data suggests that the use of cotton and soybean is dependent on a relative lack of other options in the landscape.

Our bees largely foraged locally (median communicated foraging distance = 0.702 km), with some long‐range recruitment indicating as far as ~8.3 km. While honey bees can fly several kilometers from the hive for resources, they will not typically do so unless resources near the hive are extremely scarce (Couvillon, Riddell Pearce, et al., 2014; Seeley, 1994) or if a highly rewarding alternative suddenly becomes available (Beekman & Ratnieks, 2000). A study that investigated distance over time, as indicated by the waggle dance, reported that the maximum foraging distance for bees in Southern England was ~6 km (Couvillon et al., 2014a, 2014b). Therefore, an 8 km dance should be viewed as an interesting and unusual event. These long‐distance foraging events mostly occurred in the months of May and June in 2018 and 2019, which may be due to the abnormally dry conditions in the first quarter of 2018 and in June of 2019.

The distance bees travelled for resources also varied by month and year, a trend observed in other studies and largely driven by season, major blooms, and dominant landscape features (Beekman & Ratnieks, 2000; Couvillon et al., 2014a). For our study, the shortest foraging distance, when forage is most available, occurred in July, likely due to surrounding row crops blooming concurrently. Foraging distance was relatively consistent throughout the rest of the foraging season (Figure 3). June 2019 represents one exception to this general rule, where our honey bees recruited to a median foraging distance of 1.3 km, representing a 124.6% increase from the previous year's June. Although we cannot determine the exact reasons, anecdotally we think that drought, and the related loss of two hives, contributed to these distances. Rainfall events during that month were largely seen during the first week, whereas most of the rest of the month (c. 60%) was without rainfall. Drought could represent an additional stressor that impacts available forage or forage quality through a reduction in nectar or pollen available, subsequently impacting overall colony health (Le Conte & Navajas, 2008; Minckley et al., 2013). Perhaps because of the challenging conditions, we had two hives die in early July 2019, and although they were quickly replaced with additional observation hives, there was a small gap in our data that overlapped with the bloom of corn (Figure 1), which already possesses a relatively short full bloom period. These colony deaths and the few days it took to replace them makes the bees’ use of corn even more impressive: corn accounted for 8% and 11% of the landscape, and approximately 9% and 16% of recruitment indicated the fields during bloom (Table 2, Figure 5). After a distance correction, we see that honey bees have a definite preference for corn relative to other fields during July (Figure 7).

About 35% of major food crops are insect pollinated (Klein et al., 2007). Largely because of this reliance in pollinator‐dependent crops, flower visiting insects are estimated to provide c. $200–250 billion worldwide in critical benefits to agricultural ecosystems (Gallai et al., 2009). Currently the demand for food crop pollination is outpacing the availability of pollinators (Aizen & Harder, 2009b; Garibaldi et al., 2011), as approximately 41% of insect species are declining globally (Hallmann et al., 2017; Kosior et al., 2007; Sánchez‐Bayo & Wyckhuys, 2019). Of these insect species, major classes of pollinators, such as Hymenoptera and Lepidoptera, are some of the most affected (Berenbaum et al., 2007).

Overall, our honey bees foraged in rows crops of peanut, corn, and cotton much more than initially predicted, especially given that the select row crops are not pollinator‐dependent, are generally poor nectar sources, were not overly represented in the landscape (Table 1), and can present a cumbersome challenge in pollen transport in the case of cotton (Jones & McCurry, 2012; Vaissière & Vinson, 1994; Vansell, 1944). Although our study is limited to one study site, and its location on a mixed crop research farm concentrated multiple crops within the immediate vicinity, it nevertheless presents the tracking of honey bee foraging through waggle dance decoding that spanned two full foraging years. As such, it represents an advancement in our understanding of where honey bees forage in a row crop environment and suggests that, in a similar agricultural landscape, hives might also be sustained by such crops during the summer. However, several unknowns remain: just because honey bees can feed themselves on row crops does not mean that it is the ideal option for their well‐being. Future studies could determine how honey bees might exploit row crops in landscapes with greater or lesser heterogeneity, especially given that periods of forage dearth have been observed in less heterogeneous landscapes (Dolezal et al., 2019). It would also be interesting to investigate whether a diet of row crop forage might also represent an over‐exposure to agricultural chemicals, which could have important implications for pest management.

## AUTHOR CONTRIBUTIONS

**Mary R. Silliman:** Data curation (supporting); formal analysis (supporting); investigation (equal); project administration (supporting); validation (equal); writing – original draft (lead); writing – review and editing (supporting). **Roger Schürch:** Conceptualization (equal); data curation (lead); formal analysis (lead); funding acquisition (supporting); investigation (supporting); methodology (equal); software (lead); supervision (supporting); validation (lead); visualization (lead); writing – original draft (supporting); writing – review and editing (equal). **Sean Malone:** Investigation (supporting); methodology (supporting); supervision (supporting); writing – review and editing (supporting). **Sally V. Taylor:** Conceptualization (supporting); Investigation (supporting); methodology (supporting); project administration (equal); resources (equal); supervision (equal); visualization (supporting); writing – original draft (supporting). **Margaret J. Couvillon:** Conceptualization (lead); data curation (supporting); formal analysis (equal); funding acquisition (lead); investigation (lead); methodology (lead); project administration (lead); resources (supporting); software (supporting); supervision (lead); validation (supporting); visualization (supporting); writing – original draft (equal); writing – review and editing (lead).

## Data Availability

The data corresponding to this manuscript has been curated as a static dataset for use by other researchers and the public and is stored at the Virginia Tech Data Repository (https://doi.org/10.7294/19755016).
