# Corrigendum

**DOI:** 10.1002/ece3.9085

**Published:** 2022-07-05

In the recent article by Silliman et al., (2022), the visual of when corn is in full bloom was shifted in Figure 1. The correct figure is shown below: 
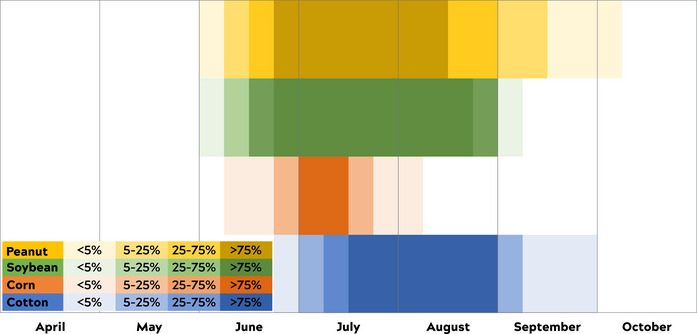


The authors apologize for the error.
